# Impact of Solute Carrier Transporters in Glioma Pathology: A Comprehensive Review

**DOI:** 10.3390/ijms24119393

**Published:** 2023-05-28

**Authors:** Filippos Anagnostakis, Michail Kokkorakis, Mariam Markouli, Christina Piperi

**Affiliations:** 1 Department of Medical and Surgical Sciences, University of Bologna, 40126 Bologna, Italy; filippo.anagnostakis@studio.unibo.it; 2 Department of Clinical Pharmacy and Pharmacology, University Medical Center Groningen, 9700 RB Groningen, The Netherlands; m.kokkorakis@umcg.nl; 3 Department of Medicine, Boston Medical Center, Boston University School of Medicine, Boston, MA 02118, USA; myriam.markouli@gmail.com; 4 Department of Biological Chemistry, Medical School, National and Kapodistrian University of Athens, 11527 Athens, Greece

**Keywords:** solute carriers, transporters, SLC, brain tumors, gliomas, tumor microenvironment, therapy

## Abstract

Solute carriers (SLCs) are essential for brain physiology and homeostasis due to their role in transporting necessary substances across cell membranes. There is an increasing need to further unravel their pathophysiological implications since they have been proposed to play a pivotal role in brain tumor development, progression, and the formation of the tumor microenvironment (TME) through the upregulation and downregulation of various amino acid transporters. Due to their implication in malignancy and tumor progression, SLCs are currently positioned at the center of novel pharmacological targeting strategies and drug development. In this review, we discuss the key structural and functional characteristics of the main SLC family members involved in glioma pathogenesis, along with their potential targeting options to provide new opportunities for CNS drug design and more effective glioma management.

## 1. Introduction

Solute carriers (SLCs) are the largest family of transmembrane transporters (consisting of 439 proteins without the pseudogenes), which are divided into 65 subfamilies (60 of which have been identified in the brain). They play a crucial role in exchanging different substances such as nutrients, ions, metabolites, as well as drugs through biological membranes [1]. Most SLCs share the same protein structure consisting of 12 presumed transmembrane segments with molecular mass ranging between 50 to 100 kDa [2].

SLCs occur in multiple isoforms with each one possibly being associated with a specific cell type. These isoforms often vary in their C- or N- termini, leading to various protein–protein interactions, transport efficacy and stoichiometry, or localization [3]. Overall, 287 SLCs have been discovered in the brain, mainly located in the blood–brain barrier (BBB) and in parenchymal cells, where they facilitate secondary active transport and diffusion processes. The SLCs found in the BBB endothelial cells protect the brain from harmful substances while absorbing necessary components from the blood. On the other hand, SLCs found in the choroid plexus of the blood–cerebrospinal fluid barrier (BCSFB) regulate cerebrospinal fluid (CSF) secretion and re-absorption. Additionally, SLCs of neurons and glial cells maintain brain homeostasis and regulate drug response. Their participation in drug delivery with cell type specificity has rendered them as potential drug therapeutic targets for multiple conditions [1].

In this review, we describe the biochemical structure and physiological role of the main SLCs detected in the brain that are also involved in brain tumor pathogenesis. We further discuss the associated pathophysiological mechanisms contributing to tumorigenesis and potential targeting options to indicate new therapeutic schemes for gliomas.

## 2. Biochemical Characteristics and Physiological Role of Main SLC Family Members

Accumulating research evidence has shown that several SLCs are involved in brain pathophysiology contributing to tumor development, and they are described in more detail in this section.

### 2.1. Cation/Anion Transport

#### 2.1.1. SLC1A5

The *SLC1A5* gene is found on chromosome 19q13.3; it contains eight exons and encodes for the SLC1A5 protein, also known as Alanine–Serine–Cysteine Transporter 2 (ASCT2). It functions as a Na^+^-dependent neutral amino acid transporter on the cell plasma membrane [4]. In particular, it is responsible for the transportation of valine, alanine, and methionine into the cell and the bidirectional transportation of asparagine, glutamate, serine, and threonine [5], with cysteine as a modulator [6]. The regulation of ASCT2 expression depends on the availability of substrates, especially glutamate, which is the preferred substrate [6], leading to an increase in its expression [7].

#### 2.1.2. SLC7A11

The *SLC7A11* gene is found on chromosome 4q28.3 and encodes for the SLC7A11 protein, which is the functional subunit of the system Xc- [8]. It comprises two subunits, namely, the light chain SLC7A11 (xCT) and the heavy chain SLC3A2 [8], with the latter anchoring the former to the plasma membrane, maintaining its stability [9]. This complex functions on the cell surface as a Na^+^-independent, CL^−^-dependent anionic L-cystine/L-glutamate antiporter and regulates the uptake of cystine found extracellularly, passing on glutamate located intracellularly at a 1:1 molar ratio [8]. Cystine from extracellular sources is transported inside the cell via SLC7A11, where it is used to produce cysteine after reduction in NADPH [9], the rate-limiting precursor for the synthesis of glutathione and ferroptosis. It has also been suggested to affect malignant cancer behavior, the TME, the function of the immune system, cancer-associated syndromes, as well as the sensitivity to therapy [8]. SLC7A11 expression can be modulated and tightly controlled in transcriptional, post-transcriptional, and post-translational levels as well as through epigenetic mechanisms [9].

#### 2.1.3. SLC8A2

The solute carrier family 8-member 2 (*SLC8A2*) gene, also named the sodium/calcium exchanger 2 (NCX2), is located on chromosome 19q13.32 and consists of ten exons [10]. It is widely expressed in neuronal cells throughout the brain, however, without evidence of expression in other tissues [11]. The *SLC8A2* gene encodes the Na^+^/Ca^2+^ exchangers (NCX2), which belong to the CaCA (Ca^2+^/Cation Antiporter) superfamily;therefore, SLC8A2 contributes to intracellular Ca^2+^ homeostasis [11]. Moreover, it enables the exchange of Ca^2+^ and Na^+^ ions through the cell membrane, regulating Ca^2+^-dependent cellular processes. Furthermore, it participates in the quick decrease in the cytoplasmic Ca^2+^ levels to baseline after neuronal activation, thereby modulating synaptic plasticity, learning, and memory processes. Lastly, SLC8A2 is involved in regulating the urinary excretion of Ca^2+^ and Na^+^ [12]. Studies indicate that changes in NCX2 expression in PC12 neuronal cells occur after activation of extracellular-signal-regulated kinases 1/2 (ERK1/2), c-Jun N-terminal kinases (JNK), and p38 mitogen-activated protein kinases (MAPKs), with nerve growth factor (NGF) pharmacologically blocked or silenced [13].

#### 2.1.4. SLC9A1

The sodium–hydrogen antiporter 1 (NHE-1), commonly referred to as SLC9A1, is encoded by the gene *SLC9A1* in humans and is located on chromosome 1p36.11 and contains 17 exons. The NHE1 protein in humans consists of 815 amino acids, with a hydrophobic N-terminal membrane domain responsible for NHE transport, consisting of 500 amino acids, and a hydrophilic C-terminus located intracellularly, consisting of 315 amino acids. The mature NHE1 protein on the plasma membrane can be N- and O-glycosylated. However, glycosylation has not been associated with its function as a transporter [13,14]. The great majority of mammalian cells express NHE1. Quite often, NHE1 presents the “housekeeping” NHE isoform. NHE almost exclusively resides on the cellular surface, with the primary function to alkalize the cell by expelling H^+^ ions generated from metabolism or electrically driven acidification. Moreover, this antiporter presents the major pathway for the entry of Na^+^ ions into the cell, which along with Cl^−^ and water uptake mediate the increase in regulatory volume following cell shrinkage. NHE1 additionally partakes in cell migration [15,16,17,18]. Lastly, the absence or loss of NHE1 in the brain membranes can modulate the activity of other transporters, which, in turn, leads to enhanced neuronal excitability [18].

#### 2.1.5. SLC11A1

The SLC11A1 (solute carrier family 11 member 1 protein) was previously named NRAMP1 (natural resistance-associated macrophage protein 1) and is found in the human macrophage membrane. *SLC11A1* is situated on chromosome 2q35, spans approximately 14 kilobases, and is comprised of 15 exons. SLC11A1 is only expressed among immune phagocytes recruited after phagocytosis to the phagosomal membrane and acts as a divalent cation transporter [19]. A microsatellite polymorphism at the *SLC11A1* 5’ terminal region that has a Z-DNA-forming dinucleotide repeat has been associated with the development of infections, autoimmunity, and diabetes in humans. During the activation of macrophages, membrane translocation to the late endosomes/lysosomes of the SLC11A1 protein takes place [20]. Its primary function is to act as an antiporter, facilitating the influx of cations into the phagolysosome or cytosol, according to the respective pH gradient [21]. Additionally, it impacts the formation of the inflammasome complex and influences the pro-inflammatory interleukins, IL-18 and IL-1β secreted from macrophages. It also regulates apoptosis by controlling the activity of cytosolic nucleotide-binding oligomerization domain proteins (NODs). Loss or gain of NOD protein functions may lead to a deregulated immune response [22]. It is also assumed that transcriptional activation of *SLC11A1* may cause apoptotic events. On the other hand, transcriptional repression of *SLC11A1* could have an impact on cell survival. It may also influence immune reactions against viral vector systems [23,24,25] by coordinating the processing of antigens and proteases’ catalytic activity in the late endosomes.

#### 2.1.6. SLC12A2

The *SLC12A2* gene is located on chromosome 1p13.2 and consists of six exons. *SLC12A2* encodes for a protein known as NKCC1 (Na-K-2Cl cotransporter-1), which serves to transport and reabsorb sodium and chloride ions. In general, SLC12 family transporters mediate the transport of chloride ions along with sodium and/or potassium ions through epithelial and nonepithelial cell membranes [26]. It is largely known that With-No-Lysine (WNK) kinases can phosphorylate and activate oxidative stress response 1 (OSR1) and STE20/SPS1-related proline–alanine-rich protein kinase (SPAK) resulting in the ultimate phosphorylation and activation of NKCC1 that imports Na^+^, K^+^, and Cl^−^ in the cell [26,27,28,29,30]. The dephosphorylation of NKCC1 is mainly carried out by protein phosphatase 1 (PP1) [31,32]. NKCC1 is expressed widely in the body and is localized at Cl^−^ secreting epithelia [33], such as the salivary and sweat gland, intestine, and lung, where it mediates the electroneutral movement from the basal interstitium inside the cell of one K^+^, one Na^+^, and two Cl^−^ ions.

#### 2.1.7. SLC16A1

The *SLC16A1* gene, also known as proton-coupled monocarboxylate transporter 1 (MCT1), located on chromosome 1p13.2, has six exons, and encodes for the ubiquitous protein MCT1 [34,35]. The expression of MCT1 can be enhanced by PPAR-α (Peroxisome proliferator-activated receptor alpha), Nrf2 (nuclear factor erythroid 2–related factor 2), and AMPK (AMP-activated protein kinase) [36]. MCT1 contains a binding site for substrates in the extracellular matrix, initially binding a proton and then a lactate anion. Consequently, this leads to a conformational change in the protein which allows the exposure of the proton and lactate to the opposite area from their release. The rate-limiting step for the net transport of lactic acid is MCT1′s return to an open conformation without a bound substrate, indicating a faster exchange of a monocarboxylate from the inside of the cell to the outside than its net transport across the membrane. Lastly, SLC16A1 has been shown to induce tumor progression through metabolic modifications in the cells [37,38,39,40]. Specifically, SLC16A1 is a target of Myc oncoproteins and its high levels present a characteristic feature of human cancers with *MYC* or *MYCN* involvement [41].

#### 2.1.8. SLC30A3

The *SLC30A3* gene is located at chromosome 2p23.3 and includes 12 exons. It encodes for Zinc transporter 3 (also known as solute carrier family 30 member 3, ZnT-3) and the membrane transport protein SLC30A. ZnT-3 is a protein found in synaptic vesicles enabling the accumulation of zinc ions [42,43]. Its function in zinc transport has also been associated with the modulation of memory formation via the extracellular signal-regulated kinases signaling pathway [44]. Furthermore, ZnT-3 and ZnT-10 downregulation in vascular smooth muscles has been involved in angiotensin-II-induced cell death [43].

#### 2.1.9. SLC39A1

Zinc transporter ZIP1 constitutes a human protein, encoded by the *SLC39A1* gene [45], which is located at chromosome 1q21.3 and consists of seven exons. The cardinal role of the protein ZIP1 is the active transportation of zinc into prostate cells.

### 2.2. Amino Acid Transport

#### 2.2.1. SLC3A2

The *SLC3A2* gene is found at chromosome 11q12.3, contains 13 exons, and encodes for the SLC3A2 protein, also called CD98 or 4F2 heavy chain (4F2hc). This is a 68 kDa type II glycoprotein [5] that has a single transmembrane domain, with its N-terminus in the cytoplasm and heavily glycosylated C-terminus on the cell surface. SLC3A2 forms dimers with various light chains of nutrient transporters such as SLC7A5 and this dimerization enables SLC3A2 to act as a chaperone, facilitating the localization of the transporters to the plasma membrane. The encoded transporter also plays a role in the regulation of intracellular calcium levels and transports L-type amino acids. The role of CD98 in development is crucial since its high expression is detected in the kidneys, placenta, testis, and bone marrow. The regulation of CD98 expression is dependent upon the ubiquitin ligases MARCH1 (membrane-associated RING-CH-type finger 1) and MARCH8, as well as pro-inflammatory cytokines, since there are many ubiquitination sites in SLC3A2 and SLC7A5, with CD98 being responsible for their trafficking [6].

#### 2.2.2. SLC7A5

The *SLC7A5* gene is found at chromosome 16q24.2, contains 10 exons [6], and encodes for a 55 kDa protein serving as a functional light chain, also called Large Amino Acid Transporter 1 (LAT1) [12]. LAT1 is part of the SLC7 family, which is a subset of the larger APC (amino acid–polyamine–Organo cation) superfamily [6] and functions as a Na^+^-independent antiporter. LAT1 exhibits a high affinity to large, branched chain, and aromatic amino acids, especially leucine, phenylalanine, and tryptophan. Additionally, it is capable of transporting both D- and L-amino acid enantiomers [46]. It forms a heterodimeric amino acid transporter [6], connected with a disulfide bridge to a 4F2 heavy chain (also called CD98). This heavy chain serves as a chaperone, aiding in the recruitment of the functional subunit to the cell membrane and stabilizing it [46], but without interfering with the transport activity of LAT1. LAT1 is involved in cell proliferation and development, especially in the bone marrow, brain, testis, and placenta, where it is highly expressed. However, due to its decreased expression in the intestine and limited capacity for transportation, LAT1 does not participate in amino acid absorption from the diet. Furthermore, the ability of LAT1 to facilitate the absorption of mercury compounds may be the reason for its fetal toxicity. The regulation of LAT1 expression depends on IL-2 secretion [6], the YAP1/TAZ pathway [47], miRNAs, lnc-RNAs, promoter methylation, and glucose levels [6].

#### 2.2.3. SLC17A7

The *SLC17A7* gene is located on chromosome 19q13.33 and consists of 11 exons [48]. It encodes for SLC17A7 (BNPI/ VGLUT1) [48], a metabolic vesicular Glutamate/H^+^ exchanger, located at the synaptic vesicles [49]. VGLUT1 is mainly involved in the transport of glutamate into the synaptic vesicles, maintaining the homeostasis of the glutamatergic system. It exerts its function by allowing H^+^ to flow into the synaptic vesicles by ATPase hydrolysis, thereby increasing membrane acidity that forms a pH gradient and a corresponding membrane potential change, providing the necessary power to transport glutamate [49]. VGLUT1 has a pivotal role in the CNS through the regulation of glutamine, especially in memory, learning, and synaptic plasticity [49].

#### 2.2.4. SLC22A5

The *SLC22A5* gene is located on chromosome 5q31.1 and consists of 11 exons. SLC22A5/OCTN2 comprises the unique, ubiquitously expressed, high-affinity carnitine plasma membrane transporter. Estrogens enable the regulation of the expression of SLC22A5 [50]. Moreover, proinflammatory cytokines, such as nuclear factor-κB (NF-κB) and tumor necrosis factor-α (TNF-α) stimulate SLC22A5 expression [51]. Additionally, there is an association between the increased phosphorylation of mTOR kinase and the activation of the transcription factor STAT3, which subsequently leads to an increase in the expression of SLC22A5 [52]. SLC22A5 comprises a plasma membrane protein that is co-translationally integrated into the membrane of the endoplasmic reticulum (ER). At the ER and at the initial extracellular loop of Golgi, SLC22A5 undergoes glycosylation [53] and is subsequently transported by vesicles to the cell surface, regulated by protein kinase C (PKC) [54].

### 2.3. Glucose Transport

#### SLC2A1

The *SLC2A1* (*GLUT1*) gene is located on chromosome 1p34.2 and includes 10 exons. GLUT1 facilitates the minimal glucose uptake necessary to maintain cellular respiration. Reduced glucose levels amplify the expression of GLUT1 in cell membranes, while increased glucose levels decrease its expression [49]. There are various ways by which the GLUT1 (SLC2A1) expression is controlled at the transcriptional level. One such mechanism involves certain transcription factors, such as c-Myc (which is produced by the *MYC* oncogene) and sineoculis homeobox 1 (SIX1), which can directly activate the expression of GLUT1 as well as other glycolysis-related genes, thus leading to increased glycolysis [49]. Additionally, the expression of GLUT1 can be influenced by factors such as HIF-1α, insulin, thyroid hormone, and cancer suppressor genes [55]. The promotion of tumor angiogenesis and the upregulation of glucose metabolism genes are linked to HIF-1α. HIF-1α is activated under anaerobic conditions, binding to hypoxia response elements (HREs), which regulate the transcription of GLUT1-related genes. This causes the increased expression of GLUT1 and a higher glucose uptake in tumor cells. Hormonal regulation also has a significant impact on GLUT1 expression [55].

### 2.4. Neurotransmitter Transport

#### 2.4.1. SLC6A2 (NET)

The *SLC6A2* gene encodes for a multi-pass membrane protein, a member of the sodium/neurotransmitter symporter family. SLC6A2 is responsible for norepinephrine reuptake into presynaptic nerve terminals, thus regulating norepinephrine homeostasis [56].

#### 2.4.2. SLC6A4 (SERT)

SERT is a member of the sodium/neurotransmitter symporter family and acts as an integral membrane protein that helps transport the neurotransmitter serotonin into presynaptic neurons from synaptic spaces in a sodium-dependent manner. This results in the termination of the action of serotonin and its recycling.

#### 2.4.3. SLC18A2

The *SLC18A2* gene encodes for a transmembrane protein that functions as a monoamine transporter of dopamine, serotonin, norepinephrine, and histamine. It mainly transports amine neurotransmitters into synaptic and secretory vesicles [57]. Polymorphisms of this gene have been associated with neurological and psychiatric diseases, including schizophrenia and bipolar disorder [57].

## 3. Implication of the Main SLC Family Members in Glioma Pathogenesis

### 3.1. SLC Implication in the Glioma Microenvironment Formation

The complex interaction of neoplastic cells with the tumor microenvironment (TME) and cancer stem-like- and immune cells has a major impact on glioma pathogenesis [58,59], with SLCs themselves appearing to play an important role in this interaction.

SLC3A2 is upregulated in gliomas and GBs [60]. Its levels show a positive correlation with increased CD4^+^ T cell and dendritic cell infiltration and a negative correlation with CD8^+^ T cell infiltration, resulting in the failure of killing cancer cells [60]. The transportation of essential amino acids (EAAs) and the activation of mTORC1, which are essential for tumor growth, are facilitated by a single CD98^−^ LAT1 light chain [61]. Moreover, the overexpression of SLC3A2 is thought to be independent of the K27M mutational status [61]. While LAT1 could serve as a more appropriate marker for glioblastoma stem cells (GSC) compared with CD98hc, the aggressiveness and malignancy of CD98^+^ glioma cells can significantly be reduced by suppressing CD98 expression [61].

LAT1 (SLC7A5) is predominantly situated in the central region of the tumor [46] and in proximity to the vascular endothelium [62]. At the brain–tumor interface, with increased BBB permeability, LAT1 is significantly increased at the capillary level, which indicates its primary involvement in angiogenesis and possibly in the immune cell infiltration process. This implies that LAT1 activity may be critical for cancer cell invasion into normal brain tissue [46].

LAT1 is further found in T cells and macrophages, as well as other peripheral immune cells, and numerous studies have demonstrated its vital role in immune system regulation in response to pathogens and chronic inflammatory diseases [46], while it is also implicated in angiogenesis, as mentioned above [63]. Moreover, overexpression of LAT1 has been observed in glioma spheres retrieved from GB patient biopsies, resulting in enhanced intracellular absorption of tryptophan through LAT1. One suggested mechanism that GB cells may use to proliferate and evade immune surveillance relies on the conversion of tryptophan into L-Kyn rather than serotonin, which inhibits the protein phosphatase PP2A and promotes cell growth and proliferation. Interestingly, treatment with the non-selective L-type amino acid transporter inhibitor, BCH, was shown to significantly reduce L-Leu uptake in glioma cell lines that express LAT1 and inhibit cell proliferation in a dose-dependent manner [46,63].

Regarding immune cell function regulation, SLC7A11 expression has also been shown to be essential. Specifically, SLC7A11 upregulation in tumor cells was demonstrated to be associated with increased resistance to temozolomide or cisplatin chemotherapy treatments. In contrast, studies have demonstrated that the deactivation of SLC7A11 by using small interfering RNA or pharmacological inhibitors such as sulfasalazine increases the sensitivity of cancer cells to proteasome inhibition [64].

### 3.2. SLC Implication in Glioma Proliferation and Progression

It has been previously demonstrated that there is a significantly positive correlation between LAT1 and Ki67, as well as a correlation between LAT1 overexpression and poor patient survival [62]. The increase in LAT1 expression in cancer cells facilitates the influx of amino acids, which can aid in the regulation of several signaling pathways and induce the activation of the mechanistic target of rapamycin (mTOR) kinase, an important nutrient sensor that facilitates tumor proliferation [46].

When it comes to GB in particular, the functional heterodimer of CD98-LAT1 (necessary for LAT1 localization to cell surface membrane) was shown to be overexpressed, enabling the increased nutritional demand necessary for cell growth. Additionally, its expression is correlated with enhanced blood cell formation and metastasis. L-leucine was shown to switch on LAT-1 in cells and enable its uptake due to its high affinity for the transporter [65]. Moreover, a positive association of LAT1 with Ki67 and increased LAT1 expression with worse survival has been demonstrated [62]. Hypoxic conditions induce HIF-2α recruitment to the proximal promoter of the *Slc7a5* gene, upregulating its expression in differentiated neuronal cells. In recurrent glioma cases, an increased 18F-FAMT, 18F-FBPA, and 11C-MET uptake has been observed [47]. Collectively, these findings suggest that LAT1-expressing cells possess stem-cell-like characteristics and a significant tumorigenic potential [61].

SLC7A11 has also been involved in the promotion of tumor proliferation by enabling the removal of lipid peroxides and inhibiting ferroptosis. This occurs through ASCL4-dependent (via GPX4) and -independent (via ALOX12) pathways. Collectively, overexpression of SLC7A11 was shown to cause tumor cell dependence on both glutamine and glucose, although the mechanisms for this dependence may vary [9]. SLC7A11 expression is adaptively elevated to mitigate ferroptosis-induced lipid ROS, while ferroptosis itself is induced by various stimuli through SLC7A11 inhibition. Depleting SLC7A11 expression hampers tumor sphere formation and increases oxidative stress [8]. Cancer cells typically experience high levels of oxidative stress, and their need for antioxidant defense exceeds what can be supplied through intracellular cysteine produced de novo or through protein catabolism. As a result, tumor cells mainly depend on cysteine uptake from the extracellular compartment through nutrient transporters [9]. Finally, SLC7A11 not only affects redox status and ferroptosis sensitivity by importing cystine, but also influences the TME by exporting glutamate.

Studies on GB have further shown that the cell cycle progression and in vitro proliferation of U87MG glioma cells is not affected by SLC8A2. On the contrary, SLC8A2 inhibits the migration of U87MG cells and their invasion, as well as the expression of related genes. A research study demonstrated a significant decrease in urokinase-type plasminogen activator (uPA), its receptor uPAR, and MMP-2/9 mRNA levels in U87MG cells expressing SLC8A2 compared with U87MG not expressing SLC8A2 [10]. Specifically, U87MG migration and invasion are inhibited by SLC8A2 through the signaling axis of ERK1/2-NF-κB-MMPs/uPA-uPAR. Moreover, SLC8A2 may inhibit the Wnt/β-catenin pathway through a decrease in COX-2 levels, while overexpression of SLC8A2 was shown to inhibit the nuclear-β-catenin protein levels. The progression to malignant glioma is also closely related to high levels of MMPs and uPA/uPAR. It has been previously demonstrated that silencing of MMP-9, uPA, and uPAR leads to inhibition of glioma cell invasion.

Another study demonstrated that the in vivo tumorigenicity of U87MG cells was inhibited by SLC8A2, confirming its role as a GB tumor suppressor. Additionally, the migratory and invasive properties of U87MG cells were decreased by SLC8A2, mainly through ERK1/2 inactivation, inhibition of nuclear translocation and NF-κB DNA binding activity, as well as reduction in the uPA-uPAR system and MMPs levels. Moreover, it was established that SLC8A2 may be a negative regulator of U87MG angiogenesis [66].

Earlier studies have shown that downregulation of SLC8A2 resulted in reduced expression of HIF-1α, COX-2, MMP-2/9, and VEGF121/165/189, while also inhibiting ERK1/2 and Wnt/β-catenin signaling [16]. These data indicate a potential suppression of blood vessel formation driven by SLC8A2-mediated suppression of vascular mimicry. Moreover, the study suggests that SLC8A2 is possibly a common regulator of endothelium dependent and non-dependent U87MG cell angiogenesis, ultimately affecting glioma angiogenesis [10].

A study investigating the mRNA expression of SLC9A1 (NHE1) in CGGA and TCGA databases detected elevated mRNA levels of SLC9A1 among high-grade gliomas. SLC9A1 expression was found to be highly upregulated in mesenchymal glioma subtypes and in wild-type isocitrate dehydrogenase (IDH)1/2 GB. Elevated SLC9A1 mRNA levels in gliomas were correlated with reduced survival. This could potentially be explained through the promotion of extracellular matrix remodeling and angiogenesis. High mRNA expression of SLC9A1 was related to increased numbers of tumor-associated macrophages. In mouse glioma models, HOE642, an NHE1 inhibitor, was shown to decrease glioma volume and invasion, and increase overall survival. Additionally, NHE1 protein blockade promoted an immunogenic tumor microenvironment by inducing the accumulation of CD8 T cells, enhancing interferon-gamma (IFN-γ) expression, and rendering animals sensitive to anti-PD-1 treatment [66]. Another study demonstrated that NHE1 is an upstream factor of the extracellular signal-regulated kinase (ERK) and a downstream target of the BRAFV600E mutation, while a positive feedback loop between NHE1-ERK phosphorylation under the regulation of the BRAFV600E mutation seems to contribute to GB cell proliferation and invasion [67]. The proliferative and invasive properties of BRAFV600E-mutant and -wild-type GB cells were suppressed by an NHE1 and/or BRAFV600E inhibitor, with the combination of both resulting in more effective inhibition, suggesting a promising new therapeutic option for GB, especially when BRAF is mutated [67]. Although the abovementioned findings show that NHE1 might be a novel treatment target against brain tumorigenesis and progression, there are no clinical studies to date evaluating the pharmacological inhibition of NHE1 protein in brain tumors [68].

In addition, GB cells have been shown to exhibit significantly elevated Cl^-^ levels in comparison with low-grade gliomas and normal cortical cells [9]. This increased chloride content has previously been correlated with enhanced NKCC1 and reduced K-Cl co-transporter function [69,70]. Moreover, cell proliferation has been linked to Na-K-2Cl co-transporter function in GB [71]; NKCC1 upregulation in human GB is correlated to tumor grade, and its suppression hinders tumor invasion [69,72]. The abnormal neuronal component of GB is characterized by increased NKCC1 immunoreactivity, while upregulated neuronal NKCC1 was atypical in the perilesional zone of tumor tissues. Furthermore, increased neuronal NKCC1 expression in phGB suggests the presence of aberrant and immature neuronal cells [69]. Another study demonstrated that there was no variation in SLC12A2 expression between SF8628 and PBT24 cell controls [73]. However, treatment of PBT24 cells with valproic acid (VPA) upregulated SLC12A2, but not in SF8628 cells, although the mechanism behind this effect remains unclear. Additionally, treatment with temozolomide (TMZ) was found to upregulate NKCC1 in both PBT24 and SF8626 cells. WNK kinase–protein phosphorylation has been previously associated with TMZ-induced NKCC1 activation [74,75,76]. Moreover, the reduction in chloride content in glioma cells is caused by the inhibition of NKCC1 function, and other mechanisms of chloride transport mediate its efflux from the cell [73,76].

*SLC17A7* has been identified as a bivalent gene in GB [77] with H3K4me3 and H3K27me3 mark co-occupancy [78], allowing chromatin to acquire both active and suppressed transcriptional states throughout the cell cycle [79]. In more detail, it acts as a bivalent tumor suppressor that inhibits GB proliferation, migration, and invasion [77]. In addition, the silencing of the oncogenic H3K9me3 methyltransferase SETDB1, which reduces cell viability and survival, appears to significantly increase SLC17A7 mRNA levels in pediatric high-grade gliomas, further confirming its tumor suppressive role [80]. SLC17A7 and glutamatergic signaling has been shown to be associated with glioma-related seizures and appears to be overexpressed in brain tissue samples of glioma patients with seizures [81].

Abnormal SLC18A2 expression may also affect GB cells, since neuronal activity promotes glioma formation through autocrine, non-synaptic, as well as synaptic paracrine mechanisms using functional synapses between glioma cells and neurons, inducing tumor proliferation and progression [82].

The expression of SLC30A3 appeared to be significantly decreased in cell lines of GB [83]. The same study reported that by restoring the expression of SLC30A3, a notable inhibition of GB cell growth and progression of the cell cycle was apparent, as well as a downregulation of N-cadherin, Snai1, and Slug proteins, indicating EMT inhibition in GB cells. Moreover, in vivo experiments showed that overexpression of SLC30A3 inhibited GB growth and metastasis. Specifically, HDAC1 was found to suppress the expression of SLC30A3, and its overexpression reversed the repressive effects of SLC30A3 on the malignant phenotype of GB cells. Activation of the MAPK pathway by SLC30A3 was also observed, leading to the suppression of the malignant behavior of GB cells. Therefore, potential epigenetic targeting of SLC30A3 by HDAC1 may present a promising therapeutic option for glioblastoma [83].

A recent study has also revealed the crucial role of SLC39A1 in the development of gliomas [45]. The analysis of bioinformatics data indicated that SLC39A1 expression was increased in glioma samples and that higher SLC39A1 levels were predictive of poorer survival rates. Univariate and multivariate analyses indicated that SLC39A1 was an independent indicator of unfavorable glioma prognosis [45]. Furthermore, SLC39A1 expression was significantly associated with pathological and clinical parameters, including tumor grade, IDH mutation, and 1p19q codeletion status. The in vitro experimental findings showed that SLC39A1 increased glioma cell proliferation, inhibiting apoptosis, and is possibly linked to the upregulation of MMP2/MMP9 [84]. Additionally, SLC39A1 might influence immune cell infiltration in the glioma TME. These results are suggestive of the novel prognostic biomarker properties of SLC39A1, as well as its potential as a target for glioma management.

### 3.3. SLC Implication in Glioma Cell Metabolism

Metabolic alterations in brain tumor cells have been gaining increasing research attention since they play a crucial role in the regulation of tumor development and progression [85], and SLCs in particular seem to significantly influence glioma cell metabolism.

SLC1A5 overexpression has been linked to multiple forms of cancer, including glioblastoma (GB) [86]. In GB, the pro-oncogenic c-Myc protein regulates the expression of SLC1A5 [87]. In addition, ASCT2 overexpression shifts cellular metabolism from utilizing glucose to glutamine pathways, favoring tumor progression. The presence of glutamine and its continuous supply interfere with the regulation of ASCT2, increasing its expression. Therefore, blocking ASCT2 may hinder tumor growth by disrupting the influx of glutamine [7].

Serotonin has been shown to drive GSC maintenance and subsequently glioblastoma progression [88]. Its uptake into glioma cells has been shown to occur through a SLC6A4/SERT-dependent mechanism [89,90]. Interestingly, glioma cell treatment with TNF-α enhances SERT-dependent serotonin uptake and also seems to activate the MAPK signaling pathway, whereas pre-treatment with the MAPK inhibitor SB203580 attenuates the TNF-α-mediated stimulation of serotonin transport [89].

SLC16A1 expression was shown to be increased in high-grade gliomas compared with healthy controls and low-grade gliomas [91]. Patients with SLC16A1 upregulation were shown to have a worse prognosis compared with those with the equivalent downregulation. Previous studies have shown that SLC16A1 expression in pseudopalisading cells in the hypoxic region of GB and its knockdown reduced invasion of GB via the transforming growth factor-β (TGF-β) signaling [92,93]. SLC16A1 was demonstrated to also regulate lactate and H^+^ uptake and was enhanced in neuroblastomas that generate high amounts of lactic acid. SLC16A1 downregulation led to increased intracellular pH in endothelial cells derived from pluripotent stem cells [94].

SLC22A5 expression was shown to be upregulated in glioma cells, and its plasma membrane levels varied [95], with different SLC22A5 expression levels revealing a correlation between the rate of fatty acid oxidation (FAO) and the transporter level, as well as the carnitine transport. The same study demonstrated that chemotherapeutic-based drug inhibition of carnitine transport with agents such as vinorelbine and vincristine inhibited FAO, which was further intensified by etomoxir, a carnitine palmitoyltransferase 1 (CPT1) inhibitor. This, in turn, resulted in reduced viability and increased apoptosis in glioma cells. Glioma cell survival was additionally affected either by silencing or upregulation of SLC22A5 levels in an FAO-dependent manner. These findings indicate that glioma cell survival is heavily dependent on both FAO and SLC22A5 activity and that CPT1 and SLC22A5 comprise potential treatment targets [95].

**Figure 1:**
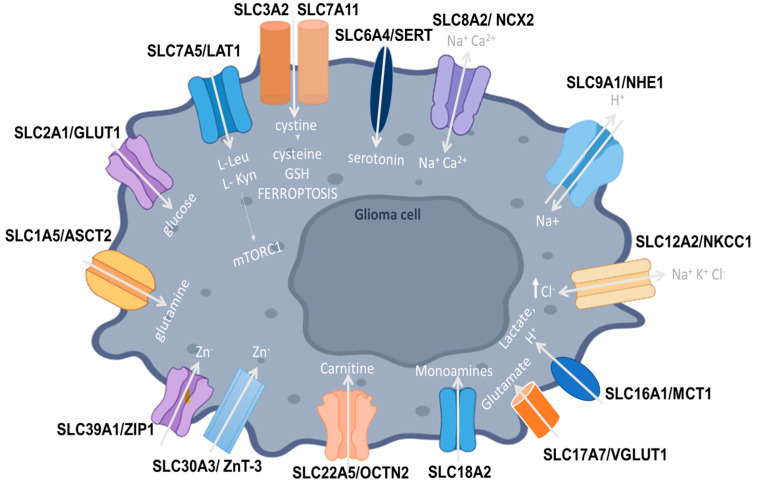
SLC1A5 (ASCT2) is a neutral amino acid transporter that shifts GB cellular metabolism from glucose to glutamine pathways, favoring tumor progression. SLC2A1 (GLUT1) increases glucose uptake in tumor cells. SLC3A2 is upregulated in glioma cells and forms dimers with various transporter light chains, such as SLC7A5 (LAT1), acting as a chaperone to facilitate their localization to the plasma membrane. LAT1 functions as an antiporter facilitating the influx of leucine, phenylalanine, and tryptophan, enhancing the activity of mTOR kinase in favor of tumor proliferation. GB cells also convert tryptophan into L-Kyn to proliferate and evade immune surveillance. SLC7A11 is the functional subunit of system Xc- that also includes the SLC3A2 heavy chain and functions as a L-cystine/L-glutamate antiporter, regulating cystine uptake that is then used to produce cysteine, the rate-limiting precursor for the synthesis of glutathione and ferroptosis. SLC6A4 (SERT) is responsible for serotonin uptake into glioma cells. SLC8A2 (NCX2) is a Na^+^/Ca2^+^ exchanger (NCX2), regulating Ca2^+^-dependent cellular processes. It inhibits invasion and migration in GB cell lines, acting as a tumor suppressor. SLC9A1 (NHE-1) transports Na^+^ ions into the cell, also promoting ECM remodeling, cellular migration, and angiogenesis. SLC12A2 (NKCC1) is a Na-K-2Cl cotransporter-1, transporting Cl^−^ ions through the plasma membrane, with high-grade GB cells having significantly enhanced NKCC1 activity and thereby elevated Cl^-^ levels. SLC16A1 (MCT1) allows for the transport of protons and lactate and enhances tumor progression through cellular metabolic modifications. SLC17A7/VGLUT1, a glutamate transporter, acts as a bivalent tumor suppressor that inhibits GB proliferation, migration, and invasion. SLC18A2 is responsible for the transport of monoamine neurotransmitters and its abnormal expression may induce glioma proliferation. SLC22A5 (OCTN2) comprises the high-affinity carnitine transporter, with SLC22A5 being upregulated in glioma cells and different SLC22A5 expression levels being correlated with FAO rates and carnitine transport. SLC30A3 (ZnT-3) aids in the accumulation of zinc ions but appears to be significantly decreased in GB cell lines. SLC39A1 (ZIP1) actively transports zinc into cells and its expression is enhanced in glioma tissues, favoring tumor progression.

All main SLC transporters implicated in glioma pathogenesis are shown in Figure 1.

## 4. Therapeutic Targeting of SLCs

SLCs mediate the transport of a wide range of solutes in CNS, being involved in brain tumor pathology and participating actively in the uptake, metabolism, and excretion of drugs. Due to these properties, SLCs constitute promising drug targets and some family members have already been therapeutically exploited in gliomas (Table 1).

More specifically, the ER stress-inducing drug, 2-deoxy-D-glucose (2-DG), which targets tumor cells through GLUT1, appears to potentiate radiation responses in GB [96]. With respect to SLC3A2 involvement in gliomas, polyamine synthesis has been detected as enhanced in the pediatric type of diffuse intrinsic pontine gliomas (DIPG), thus increasing sensitivity to difluoromethylornithine (DFMO), an irreversible inhibitor of ornithine decarboxylase 1 (ODC1), which is the rate-limiting step in polyamine synthesis. DIPG cells have been shown to upregulate the polyamine transporter SLC3A2, in order to compensate for ODC1 inhibition. Additionally, AMXT 1501, a polyamine transporter inhibitor was shown to reduce DIPG polyamine uptake and its combination with DFMO was further demonstrated to exert potent in vitro effects and extend survival in three DIPG orthotopic animal models. Therefore, these data suggest the promising effects of targeting polyamine uptake and synthesis as a potential therapeutic scheme for the devastating pediatric DIPGs and high-grade gliomas since the combination of AMXT 1501 and DFMO treatment effectively increased cell death and improved survival [97].

When it comes to NET, its therapeutic targeting is useful in advanced and refractory neuroblastoma, while NET-targeting 123I-metaiodobenzaguanidine (MIBG) therapy is an option for other neuroendocrine tumors as well [98].

Moreover, LAT1 was additionally demonstrated to affect drug uptake in GB cells. Novel chemotherapeutic approaches include the LAT1-mediated chemotherapy delivery and particularly WP1066-loaded liposomes with Amphi-DOPA administered intravenously, in combination with dendritic cell (DC)-targeted DNA vaccination. A DNA vaccine employing survivin, a known GB antigen, was used [98]. This combination significantly expanded the overall survival rate (by approximately 60%) of mice bearing orthotopic GB [99]. In another study, the uptake of nanoparticles loaded with doxorubicin and coupled with L-Phe by C6 glioma cells was achieved through the LAT1 transporter, ultimately leading to cancer cell cytotoxicity in an ex vivo setting [46]. Furthermore, the 3CDIT is a novel derivative of an amino acid that can cross the blood–brain barrier and target glioma tumor cells by interacting with the overexpressed LAT1 transporter [46].

Research studies have also shown that targeting the ribozyme-controlled HSVtk gene (human herpes simplex virus thymidine kinase type 1 gene) by overexpressing the miR-145 can lead to significant downregulation of various “metastasis-related genes”, including *LAT1* [46]. On the other hand, treating medulloblastoma cells with the LAT1 inhibitor JPH203 over an extended period leads to cellular adaptation but not resistance, which ultimately impairs cell proliferation, survival, and migration [100]. JPH203 has the ability to inhibit the mTORC1 pathway, which can reduce the proliferation and survival of cancer cells, ultimately resulting in an effective and complete anticancer effect [46]. Finally, the phenylalanine derivative labeled with astatine, 211At-PA, targets system L amino acid transporters and was shown to suppress tumor proliferation in C6 and GL-261 glioma models. These derivatives have a short half-life and need to be distributed promptly to be effective in management, making them a potentially valuable option for non-responder patients with highly invasive glioma [101].

When it comes to radiotherapy, boron neutron capture therapy (BNCT) presents a treatment method utilizing boron irradiation with neutron beams to produce antineoplastic effects in cancer cells that are characterized by LAT1 overexpression [46]. BNCT allows for the application of high-dose particle radiation selectively to tumor cells in which boron phenylalanine is accumulated, such as glioma cells where accumulation increases with increasing tumor grade [102]. Combining BNCT with gene therapy is advantageous for treating tumors that have low expression of LAT1 [103]. The use of BSH-polyR has also been explored as a boron agent for BNCT in cells with low LAT1 expression and has been shown to effectively trigger BNCT-dependent apoptosis, particularly in CD44 high-expressing cells since the CD44 protein acts as a major target of BSH-polyR [104].

On the other hand, SLC8A2 is possibly a common regulator of endothelium-dependent and non-dependent U87MG cell angiogenesis, ultimately affecting glioma angiogenesis. Due to its combined endothelium-dependent and -independent inhibitory properties, SLC8A2 presents an emerging and promising target for anti-angiogenic therapy in the management of GB [10].

Moreover, a diuretic drug, bumetanide was shown to inhibit NKCC1 in GB, resulting in reduced migration of glioma cells in vitro, decreased invasion of peritumor tissue in vivo, and increased tumor cell apoptosis [105,106].

Finally, glioma cell survival has been shown to be heavily dependent on both fatty acid oxidation (FAO) and SLC22A5 activity because carnitine, which is required for FAO, is delivered to the cell by SLC22A5, which is upregulated in gliomas. Inhibition or overexpression of SLC22A5 in vitro was shown to reduce the survival of glioma cells through FAO modulation [107]. Additionally, a therapeutic approach targeting both SLC22A5 and carnitine palmitoylotransferase 1 (CPT1), which mediates the formation of acylcarnitine from L-carnitine required for FAO, was demonstrated to reduce glioma cell proliferation and provoke persistent apoptosis [107]. In agreement, the combination of a chemotherapeutic agent inhibiting carnitine uptake transported by SLC22A5, along with the CPT1 inhibitor etomoxir, exhibited a synergistic effect with a more robust inhibition of FAO, decreasing survival and enhancing glioma cell death [95].

**Table 1:** Main SLC family members involved in glioma pathogenesis with targeting potential.

SLC	Carrier Type	Role in Gliomas	Therapeutic Targeting	Clinical Trials	References
SLC1A5 (ASCT2)	Na^+^-dependent neutral amino acid transporter	Shifts tumor cell metabolism from glucose to glutamine pathways			[4,86,108]
SLC2A1 (GLUT1)	Glucose uptake transporter	Increased expression in tumor cells that facilitates higher glucose uptake	2-DG potentiated the effects of radiation therapy in GB	I (NCT00096707)	[55]
SLC3A2	Acts as a chaperone, facilitating the localization of the transporters to the plasma membrane	Negatively correlates with CD8^+^ T cell levels; therefore, it is associated with decreased cancer cell killing Transports essential amino acids and activates mTORC1, leading to tumor growth	DFMO and AMXT 1501 co-administration in DIPG and high-grade glioma increased cell death and improved survival	I/II (NCT05500508)	[60,108]
SLC6A2 (NET)	Norepinephrine transporter		NET-targeting 123I-metaiodobenzaguanidine (MIBG) therapy		[98]
SLC6A4 (SERT)	Monoamine serotonin transporter	TNF-α increases SERT-dependent serotonin uptake into glioma cells and activates the MAPK signaling pathway			[89]
SLC7A5 (LAT1)	Na^+^-independent antiporter, heterodimeric amino acid transporter	Angiogenesis and cancer cell invasion Facilitates amino acid influx and mTOR activity, aiding in tumor proliferation Evasion of immune surveillance	IV WP1066-loaded liposomes of Amphi-DOPA, combined with DC-targeted DNA vaccination in vivo Nanoparticles loaded with doxorubicin and coupled with L-Phe -3CDIT -HSVtk gene targeting through miR-145 over-expression -JPH203 LAT1 inhibitor -211At-PA -BNCT and BSH-polyR use	I (UMIN000016546)	[6,47]
SLC7A11	Na^+^-independent, Cl^−^-dependent anionic L-cystine/L-glutamate antiporter	Removes lipid peroxides and inhibits apoptosis, aiding in tumor proliferation Immune cell regulation Resistance to chemotherapy			[8,9,64,109]
SLC8A2 (NCX2)	Na^+^/Ca^2+^ exchanger	Acts as a tumor suppressor, inhibiting tumor cell invasion Negatively regulates angiogenesis			[10,12]
SLC9A1 (NHE1)	Na^+^-H^+^ antiporter-1	Promotes ECM remodeling and angiogenesis Related to the accumulation of tumor-associated macrophages			[13,15,16,17,110]
SLC11A1 (NRAMP1)	Antiporter, facilitating the influx of cations into the phagolysosome or cytosol				[19,20]
SLC12A2 (NKCC1)	Na-K-2Cl cotransporter-1	Linked to GB cell proliferation Associated with aberrant and immature neuronal cells	Bumetanide reduced GB migration and invasion		[26,30,72,106]
SLC16A1 (MCT1)	Proton-coupled monocarboxylate transporter	Increases glioma cell invasion and mitotic activity			[35,91]
SLC18A2	Monoamine/neurotransmitter transporter	Promotes glioma formation			[82]
SLC22A5 (OCTN2)	Carnitine plasma membrane transporter	Correlated with FAO rates and carnitine transport Associated with glioma cell viability and reduced apoptosis	Combined SLC22A5 and CPT1 targeting Chemotherapy inhibited carnitine uptake and etomoxir		[50,95,107]
SLC30A3	Zinc transporter	Inhibits GB cell growth and cell cycle progression Inhibits EMT in GB Represses GB malignant phenotype			[83]
SLC39A1	Zinc transporter	Promotes glioma cell proliferation Influences immune cell infiltration in the glioma TME			[45,84]

## 5. Conclusions

Taken altogether, the brain contains a plethora of SLCs with prominent roles in the allocation of many substrates. Therefore, understanding the importance that these substrates have in the brain will be facilitated by discovering the potential role of various SLC families and their implication in brain homeostasis and pathogenesis. Even though some initial progress has been made, it is of utmost importance that more SLCs comprise treatment targets or be used to enable the effective delivery of drugs through the BBB and transport them to brain cells. We are still far from the elucidation of the SLC-mediated transport system in the human brain, despite continuous efforts and ongoing research. For most centrally expressed SLCs, there is a need to elucidate their physiological activity and regulation mechanisms in order to comprehend their involvement in disease onset and progression as well as in future drug design. Hence, a deeper understanding of SLCs and the aforementioned aspects will enhance drug discovery and probably enable risk stratification.

To this end, a previous study performed a dual track screening of a small-molecule neuropharmaceutical library to detect drugs penetrating the BBB that also present a high affinity for SLC-mediated transport, suggesting a new model for the discovery of novel small-molecule CNS drugs [111]. Another study analyzed transporter-related drug design to report on advancements in pharmacokinetic properties and drug toxicities, highlighting the importance of transporter recognition of the N-containing group of substrates [112].

Herein, we selected the main SLCs which are involved in the pathology of brain tumors to discuss their structural and functional characteristics. Our objective was to inspire the development of new drugs for brain tumors by showcasing the methods of transportation, drug screening, and transporter-focused drug design, ultimately aiming to increase the repertoire of therapeutic schemes for patients with brain tumors.

## Data Availability

Not applicable.
